# The Changes of Serum Leptin and Kisspeptin Levels in Chinese Children and Adolescents in Different Pubertal Stages

**DOI:** 10.1155/2016/6790794

**Published:** 2016-11-20

**Authors:** Hui juan Zhu, Su juan Li, Hui Pan, Naishi Li, Dian xi Zhang, Lin jie Wang, Hong bo Yang, Qinyong Wu, Feng ying Gong

**Affiliations:** ^1^Department of Endocrinology, Key Laboratory of Endocrinology, National Health and Family Planning Commission, Peking Union Medical College Hospital, Chinese Academy of Medical Sciences, Beijing 100730, China; ^2^Emergency Department, The First Affiliated Hospital of Zhengzhou University, Zhengzhou, Henan Province, China; ^3^Department of Laboratory Medicine, Peking Union Medical College Hospital, Chinese Academy of Medical Sciences, Beijing 100730, China

## Abstract

The aim of the study is to investigate the changes of serum leptin and kisspeptin levels in children and adolescents with different pubertal stages and nutritional states. A total of 647 Chinese children and adolescents were recruited, and serum estradiol, testosterone, pituitary gonadotropins, leptin, and kisspeptin levels were measured. The results showed that serum leptin levels of boys in T2 stage were the highest among the five stages, while they showed a gradual increase from T1 to T5 stage in girls and reached the highest in T5 stage (*P* < 0.05). Conversely, serum kisspeptin levels of boys were higher in T4 and T5 stages than those in T1 stage, while its levels of girls were the highest in T2 stage, 21.4% higher than those in T1 stage (*P* < 0.05). Both leptin and kisspeptin levels were positively correlated with BMI, WC, and weight in all boys and girls (all *P* < 0.05). In conclusion, kisspeptin levels were firstly found to be notably changed in pubertal stages and nutritional status in Chinese children and adolescents with a significant sexual dimorphism. Obese/overweight girls had higher kisspeptin levels, and there was a positive correlation between kisspeptin and FSH and LH and obesity-related parameters in all boys and girls.

## 1. Introduction

Sexual maturation and reproductive competence are gradually obtained through the whole process of puberty. Human reproductive endocrine system mainly consists of hypothalamus, pituitary, and reproductive glands. Pubertal onset requires the gradually increased and periodic impulsive secretion of gonadotropin-releasing hormone (GnRH), which is produced by hypothalamic GnRH neurons [1]. Many factors including hereditary factors, environmental factors, hormonal factors, body fat reserves, and nutritional state influence the onset of puberty [1]. However, the detailed mechanism by which the onset of puberty is triggered is still currently unknown.

Leptin is an adipocyte-derived hormone which is encoded by the* ob* gene. A growing body of evidences has demonstrated that leptin plays a key role in the regulation of body fat mass through suppressing the appetite and increasing energy expenditure by acting on the hypothalamus [2]. Recent studies found that leptin also exerted a critical role in regulation of reproductive functions. For example, leptin deficiency which was derived from* ob* gene mutation can lead to hypogonadotropic hypogonadism in addition to severe obesity in humans [3]. Leptin treatment can correct the reproductive failure of* ob/ob* mice, which cannot be reversed through weight loss by fasting [4] and accelerate the onset of puberty in normal weaning female mice without notable effects on body weight [5].

Kisspeptin is derived from a 145-amino acid precursor which is encoded by Kiss1 gene and is mainly expressed in hypothalamus and placenta [6]. Kisspeptin is able to activate the G protein-coupled receptor (Kiss1R). Recent studies have revealed that kisspeptin/Kiss1R system is involved in puberty regulation of the reproductive axis and energy metabolism. Firstly, loss-of-function mutations in kisspeptin (Kiss1) or kisspeptin receptor (Kiss1R) genes cause infertility due to lack of pubertal maturation and hypogonadotropic hypogonadism in both mice and humans [7, 8]. Activating mutation of Kiss1 or Kiss1R gene can cause central precocious puberty in humans [9, 10]. Secondly, kisspeptin administration may accelerate vaginal opening (an external sign of puberty onset) in rodents and induces gonadotropins secretion and ovulation in both rodents and humans [11–13]. Thirdly, energy deficiency reduces hypothalamic kisspeptin expression and increases Kiss1 mRNA in adipose tissue in rodents [14]. Finally, Kiss1R knockout results in impaired glucose tolerance and obesity in adult female mice [15], while kisspeptin administration may reduce food intake in rodents [16, 17].

Ma et al. [18] measured serum kisspeptin levels in 88 healthy southern Chinese adolescent girls and found it was lower in the prepubertal stage and sharply increased to the highest level in pubertal stage 2. However, to the best of our knowledge, there is no report about kisspeptin levels changes in adolescent boys, especially in Chinese population. It is still unclear whether there are some associations between serum kisspeptin and leptin in the development of puberty since leptin plays an essential role in the regulation of puberty as we all know [2, 3]. In the present study, we investigate the changes of serum kisspeptin and leptin levels in children and adolescents from Northern China in different pubertal stages (T1–T5) and normal/overweight/obesity status. Our results found that serum leptin and kisspeptin levels changed in a different, even opposite, way with pubertal stages in Northern Chinese boys and girls. Both leptin and kisspeptin were associated with obesity-related parameters and exhibited the significant gender-based differences in circulating concentrations.

## 2. Subjects and Methods

### 2.1. Study Participants and Clinical and Biochemical Measurements

A total of 647 children and adolescents (357 boys and 290 girls, ages 6–18 yr) were recruited from the elementary and middle school (from Nov. 2006 to Dec. 2006) in the suburbs of Beijing, China, and were in good health. All subjects underwent physical examination and laboratory fasting biochemical measurements. All participants had normal (age-appropriate) pubertal development. Pubertal stage was assigned according to the criteria of Tanner for breast development in females and genital development in males through physical examination by a single endocrinologist [19, 20] and also was assessed as a continuous variable using the measurement of serum concentrations of testosterone, estradiol, and gonadotropins including luteinizing hormone (LH) and follicle-stimulating hormone (FSH). Standing height and body weight were determined with a precision of 0.1 cm and 0.1 kg, respectively. Waist circumference was measured with a soft tape on standing subjects midway between the lowest rib and the iliac crest and was also determined with a precision of 0.1 cm. Body mass index (BMI) was calculated as weight (kg) divided by the square of height (m). The nutritional status of each subject was measured according to Chinese children and adolescents BMI percentile rank chart established in 2009 [21] and the criteria worldwide using BMI to judge the overweight and obese children and adolescents [22] (normal weight, BMI 5th–85th; overweight, BMI 85th–95th; obesity, BMI ≥ 95th; relative to age and gender).

Basal blood samples were taken between 0800 and 1000 h following an overnight fast. Total cholesterol (TC), triglycerides (TG), high-density lipoprotein- (HDL-) cholesterol, low-density lipoprotein- (LDL-) cholesterol, glucose, creatinine (Cr), urea, alanine aminotransferase (ALT), aspartate aminotransferase (AST), and whole blood routine for WBC, RBC, HB, and platelet and urine routine were measured by routine automated laboratory methods. Patients with chronic internal or endocrine diseases and any other diseases, which might have influenced our results, were excluded. All participants and their parents or guardians gave written informed consent before taking part in the study. All authors listed in the manuscript had access to information that could identify individual participants during or after data collection.

### 2.2. Serum Endocrine Hormones, Leptin, and Kisspeptin Assays

LH, FSH, testosterone (T), and estradiol (E2) were measured by chemiluminescence immunoassay methods (Beckman Coulter, DXI 800, USA) in our hospital. Serum leptin and kisspeptin concentrations were measured by enzyme-linked immunosorbent assay methods with commercially available kits (USCNK Life Science, Cloud-Clone Corp., USA). Interassay and intra-assay coefficients of variation were 13.5% and 5.7% for leptin and 5.8% and 5.9% for kisspeptin, respectively. The detection of kisspeptin had high sensitivity and excellent specificity according to the introduction of the kit. The recovery rates of the serum samples were in the average 90% (84–96%), and the linearity of the kit for serum sample was 80–92% in the dilution of 1 : 2, 82–94% in the dilution of 1 : 4, 97–105% in the dilution of 1 : 8, and 87–101% in the dilution of 1 : 16.

### 2.3. Statistical Analyses

Digital data were shown as mean ± SE. Statistical analyses were performed by SPSS 16.0 for Windows (SPSS Inc., Chicago, IL, USA). *t*-test and ANOVA were used separately to compare the differences between two and more than two groups. Correlation analyses and multiple regression analyses were done to explore the potential factors that could influence serum levels of leptin and kisspeptin. *P* value less than 0.05 was considered to be statistically significant.

## 3. Results

### 3.1. Biochemical, Endocrine, and Anthropometric Characteristics of All Subjects

**Figure 1:**
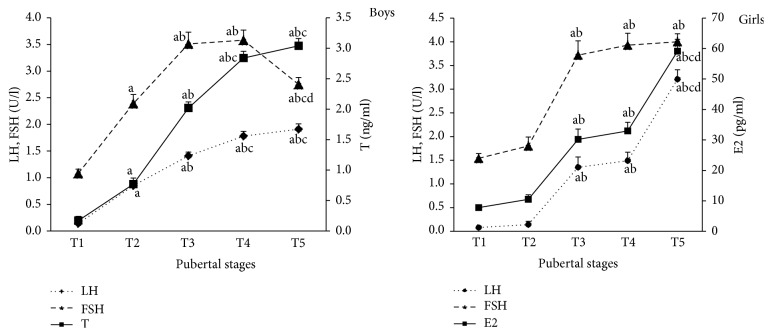
Serum LH, FSH, and T (boys) or E2 (girls) levels in different pubertal stages. (a, b, c, and d compared with T1, T2, T3, and T4, resp., *P* < 0.05).

The general characteristics of the total boys and girls in different pubertal stages were shown in Table 1. As we expected, the age, height, and weight of boys and girls all gradually increased from T1 to T5 stage and reached the highest in T5 stage (all *P* < 0.05). The age of pubertal onset of boys and girls was 12.0 ± 0.2 yr and 8.9 ± 0.2 yr, respectively. Serum LH, T, and E2 levels of boys and girls also gradually elevated from T1 to T5 stage as presented in Figure 1. For boys, serum LH and T levels in the next Tanner stage were significantly higher than those in previous Tanner stage among T1–T4 stages (*P* < 0.05), while, for girls, there were two notable increases in serum LH and E2 levels from T2 to T3 stage and T4 to T5 stage (*P* < 0.05), and there was a platform between T3 and T4 stage. In addition, there was a trend that serum T levels of obese/overweight boys were lower than those of normal weight boys from T2 to T5 stage, and they were significantly lower only in T2 stage, which represented the onset of puberty (0.22 ± 0.08 ng/mL versus 0.87 ± 0.11 ng/mL) (*P* < 0.05).

### 3.2. Serum Leptin and Kisspeptin Concentrations in Different Pubertal Stages

**Figure 2:**
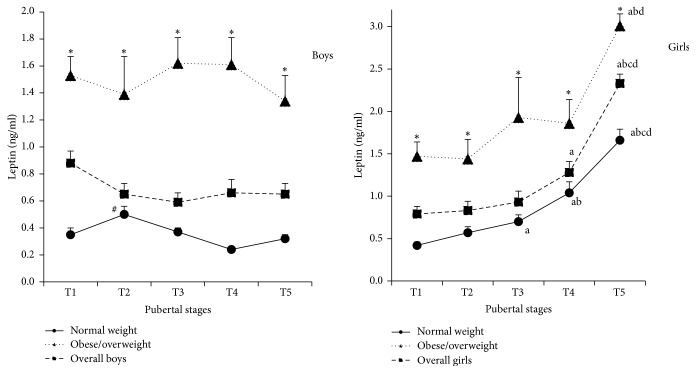
Serum leptin levels of boys and girls in different pubertal stages (boys: ^#^compared with the other four stages, *P* < 0.05; girls: a, b, c, and d compared with T1, T2, T3, and T4, resp., *P* < 0.05; boys and girls: ^**∗**^compared with normal weight children in each pubertal stage, *P* < 0.05).

As shown in Figure 2, serum leptin levels in prepubertal normal weight boys were 0.35 ± 0.05 ng/mL (T1) and immediately increased to 0.50 ± 0.06 ng/mL, the highest levels among the five Tanner stages, following the onset of puberty (T2) (*P* < 0.05), and then gradually decreased to the prepubertal levels. However, there were no significant differences in serum leptin levels of overall or obese/overweight boys among pubertal stages, although their levels of obese/overweight boys in every pubertal stage were significantly higher than those in normal weight boys (*P* < 0.05) (Figure 2). Unlike what was presented in boys, serum leptin levels in normal weight girls increased gradually from 0.42 ± 0.04 ng/mL (T1) to 1.66 ± 0.13 ng/mL (T5), which was the highest among the five Tanner stages (*P* < 0.05). The similar increasing trend of serum leptin levels was also observed in overall and obese/overweight girls from stages T1 to T5 (Figure 2). In accordance with what was presented in boys, serum leptin levels of obese/overweight girls in every pubertal stage were also significantly higher than those in normal weight girls (*P* < 0.05) as shown in Figure 2.

**Figure 3:**
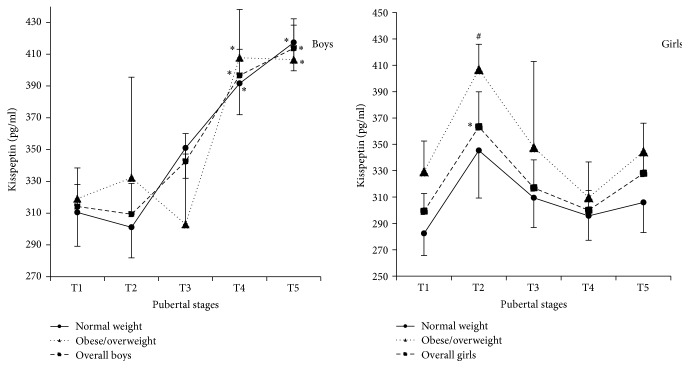
Serum kisspeptin levels of boys and girls in different pubertal stages (boys: ^**∗**^compared with T1, *P* < 0.05; girls: ^**∗**^compared with T1 and T4, *P* < 0.05, and ^#^compared with T1, T4, and T5, *P* < 0.05).

In contrast to the changes of leptin in boys and girls among the five pubertal stages, serum kisspeptin levels in overall boys continuously increased from 314.1 ± 14.0 pg/mL (T1) to 413.8 ± 14.5 pg/mL (T5) as shown in Figure 3. Serum kisspeptin levels in T4 and T5 stages were 26.3% and 31.7% higher in comparison with those in T1 stage, respectively (both *P* < 0.05). The similar change trend was also observed in normal weight and obese/overweight boys (Figure 3). However, serum kisspeptin levels in prepubertal overall girls were 299.2 ± 13.5 pg/mL (T1) and sharply increased to 363.3 ± 26.6 pg/mL (T2), which was the highest among the five pubertal stages, 21.4% higher than that in T1 stage (*P* < 0.05), and then gradually decreased to the prepubertal levels in T5 stage. The similar change trend was also observed in obese/overweight girls, showing that serum kisspeptin levels of T2 stage were the highest, 23.6% higher than those in T1 stage (*P* < 0.05) (Figure 3).

### 3.3. Comparison of Serum Leptin and Kisspeptin Levels between Boys and Girls

**Figure 4:**
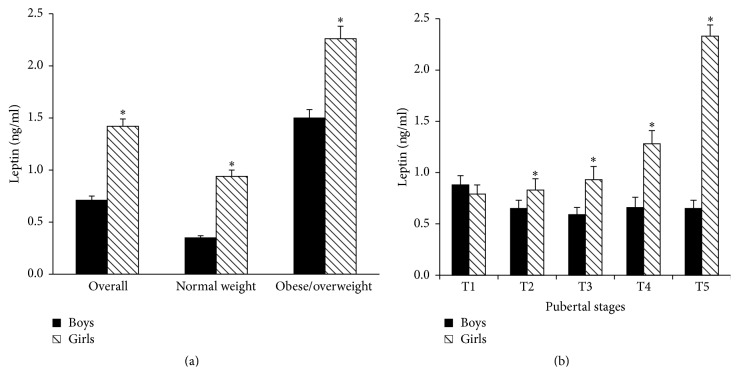
Serum leptin levelsbetween boys and girls in different nutritional states (a) and different pubertal stages (b) ((a) ^**∗**^compared with boys, *P* < 0.01; (b) ^**∗**^compared with boys, *P* < 0.05).

As depicted in Figure 4(a), serum leptin levels of girls including the overall, normal weight, and obese/overweight girls were 2.00, 2.70, and 1.50 times those of the boys in the corresponding group, respectively (all *P* < 0.01). In addition, serum leptin levels of girls from T2 to T5 stage were also significantly higher than those of boys in corresponding stage (all *P* < 0.05) (Figure 4(b)).

**Figure 5:**
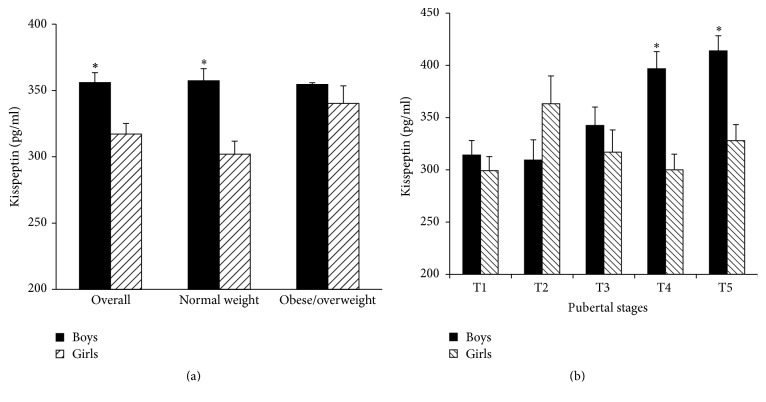
Serum kisspeptin levels between boys and girls in different nutritional states (a) and different pubertal stages (b) ((a) ^**∗**^compared with girls, *P* < 0.01; (b) ^**∗**^compared with girls, *P* < 0.05).

In contrast to the changes of leptin between boys and girls, serum kisspeptin levels in overall and normal weight girls were significantly lower than those in boys of the corresponding group (*P* < 0.01) (Figure 5(a)). The decreased kisspeptin levels were not observed in obese/overweight girls. Meanwhile, serum kisspeptin levels of girls in T4 and T5 stages were also significantly lower than those of boys, and they were 75.6% and 79.3% of boys in T4 and T5 stages, respectively (*P* < 0.01) (Figure 5(b)).

### 3.4. Comparison of Serum Leptin and Kisspeptin Levels between Normal Weight and Obese/Overweight Children

**Figure 6:**
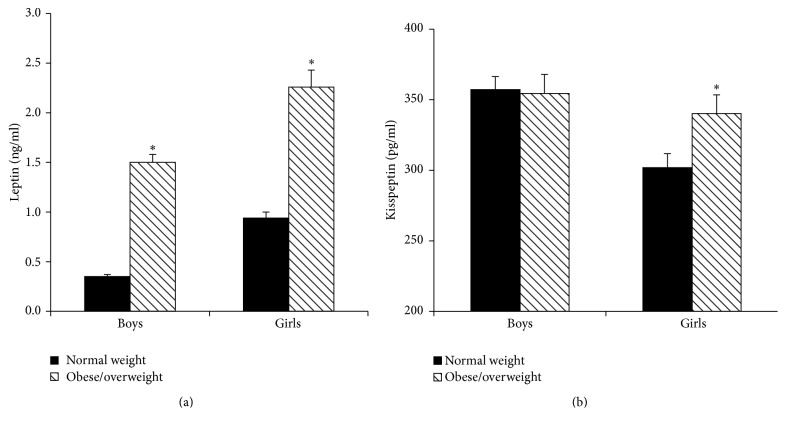
Serum leptin (a) and kisspeptin (b) levels between normal weight and obese/overweight children ((a) ^**∗**^compared with normal weight children, *P* < 0.01; (b) ^**∗**^compared with normal weight children, *P* < 0.05).

As shown in Figure 6(a), serum leptin concentrations of obese/overweight girls were 2.40 times those of normal weight girls (2.26 ± 1.19 ng/mL versus 0.94 ± 0.79 ng/mL, *P* < 0.01). Similarly, serum leptin levels of obese/overweight boys were also notably higher and were 4.29 times those of normal weight boys (1.50 ± 0.88 ng/mL versus 0.35 ± 0.27 ng/mL, *P* < 0.01). Furthermore, as we mentioned above, when the boys and girls were grouped by Tanner stage, serum leptin levels of obese/overweight group in every pubertal stage were also higher than those in normal weight group in both boys and girls as presented in Figure 2 (*P* < 0.05).

In line with the increased leptin levels in obese/overweight girls, serum kisspeptin levels in obese/overweight girls were also significantly higher than those in normal weight girls as shown in Figure 6(b) (340.2 ± 137.0 pg/mL versus 301.9 ± 133.1 pg/mL, *P* = 0.02). However, there was no significant difference in serum kisspeptin levels between normal weight and obese/overweight boys.

### 3.5. Correlation and Regression Analysis of Serum Leptin and Kisspeptin Levels with Other Clinical Items

Serum leptin levels were positively correlated with BMI, WC, weight, LDL-C, and TG and negatively related to HDL-C in both boys and girls (all *P* < 0.05) as presented in Table 2. Leptin levels were also positively correlated with E2 (*r* = 0.442), FSH (*r* = 0.200), and LH (*r* = 0.368) in girls (all *P* < 0.05) but negatively correlated with T (*r* = −0.193) and LH (*r* = −0.101) in boys (all *P* < 0.05). Additionally, serum kisspeptin levels were positively correlated with BMI, WC, weight, LH, and FSH in both boys and girls (all *P* < 0.05), and they were also positively related to age (*r* = 0.277) and T (*r* = 0.245) and negatively related to HDL-C (*r* = −0.221) in boys (all *P* < 0.05) (Table 2). On the other hand, circulating leptin levels were significantly correlated with serum kisspeptin levels in girls (*r* = 0.140, *P* < 0.05).

As depicted in Table 3, BMI, WC, and T were independent contributors to serum leptin levels in boys by using stepwise multiple regression analysis and taking serum leptin levels as the dependent variable (*R* = 0.750, *R* ^2^ = 0.562, and *P* < 0.05), while WC, BMI, and E2 were independent contributors to serum leptin levels in girls (*R* = 0.834, *R* ^2^ = 0.696, and *P* < 0.05). Meanwhile, stepwise multiple regression analysis also showed that serum kisspeptin levels were independently affected by HDL-C in boys (*R* = 0.329, *R* ^2^ = 0.108, and *P* < 0.05) and by FSH in girls (*R* = 0.225, *R* ^2^ = 0.051, and *P* < 0.05) after adjusting the age.

## 4. Discussion

To the best of our knowledge, this is the first study on serum kisspeptin levels in Chinese children and adolescents in different pubertal stages in such a sizable sample. In this study, we found that serum kisspeptin levels were significantly changed in children from T1 to T5 stage in different nutritional status with a significant sexual dimorphism. Kisspeptin levels in obese/overweight girls were significantly higher than those in normal weight girls, and they were positively correlated with weight, BMI, WC, FSH, and LH in all boys and girls.

### 4.1. Associations of Leptin with Pubertal Stages and Gender in Normal/Overweight/Obesity Status

Our study showed that serum leptin levels of normal weight boys were highest at pubertal stage 2 and then declined to prepubertal levels at pubertal stage 5, while they experienced a steady increase in normal weight girls from T1 to T5 stage and reached the highest levels at pubertal stage 5. Consistent with our results, data from Horlick et al. [23] found that circulating concentrations of leptin firstly rose in males at the early pubertal stage and then fell significantly at the later pubertal stage, whereas they continued to rise in females from Tanner stages I–V in American children. The similar phenomenon was also observed in studies performed by Blum et al. [24] and Tang et al. [25] in Germans and southern Chinese children. Additionally, Maqsood et al. [26] and El-Eshmawy et al. [27] found that there was a significant correlation between both leptin and LH and leptin and FSH in children progressing into puberty. In the present study, we found that the gradual increase of leptin from T1 to T5 in girls was consistent with the change trend of LH and FSH, and there was a positive relationship between leptin and LH and FSH. However, there was no such change trend and relationship between leptin and LH and FSH in boys. Therefore, leptin may exert the different regulatory function in the pubertal development process of boys and girls. But the exact sexual dimorphism mechanism by which leptin regulated the pubertal process needs to be figured out in the future.

It has already been illustrated that serum leptin concentrations are significantly higher in female adults than in male adults, even after adjustment for BMI and percentage of body fat [28, 29]. Similarly, our study also demonstrated that serum leptin concentrations in girls were notably higher than in boys after the onset of puberty (from pubertal stage 2 to 5), and serum leptin levels were positively correlated with estradiol in girls but negatively correlated with testosterone in boys. The similar results were also observed in the studies performed by Blum et al. [24], which showed that serum leptin levels of German girls were significantly higher than those of German boys. The following several studies would give an explanation for the gender-based differences of circulating leptin levels. Jockenhövel et al. [30] found that testosterone substitution could normalize the elevated serum leptin levels in hypogonadal men. Administration of human adipocytes with androgens clearly suppressed leptin secretion and mRNA expression in* in vitro* study [31]. In addition, one random control trials (RCT) study performed in healthy postmenopausal women showed that 17*β*-estradiol replacement for two months could significantly increase the median serum leptin levels from 17.6 *μ*g/L to 24.1 *μ*g/L without changing the percentage of body fat and body fat mass [32].

It has been well known that the most important factor that determines circulating leptin levels is body fat mass, and leptin reflects the proportion of adipose tissue under the conditions of regular eating cycles [33]. Therefore, the increased serum leptin levels were observed in obese adults, and they were positively correlated with the percentage of body fat and BMI [33]. In our present study, we also found that serum leptin levels were higher in obese/overweight children and adolescents in comparison with normal weight subjects at each pubertal stage both in boys and in girls. Serum leptin concentrations were positively correlated with BMI, WC, and body weight. These results were in concordance with previous literature reported by Horlick et al. [23], Blum et al. [24], Tang et al. [25], and Rutters et al. [34], who all demonstrated that serum leptin levels were significantly correlated with BMI, percentage of body fat mass in American, Germans, southern Chinese, and Dutch children.

More importantly, in our present study, we found serum testosterone levels of obese/overweight boys were significantly lower than those of normal weight boys in T2 stage, which represented the onset of puberty. These results implied that obese/overweight boys have more easily suffered from the delayed pubertal timing, which was in accordance with what we have observed in our daily clinical work. The lower testosterone in obese/overweight boys may be related to the increased leptin because androgens could suppress the leptin expression in adipocytes [31]. With respect to girls, the elevated serum leptin levels in obese/overweight girls and the positive relationship between leptin and estradiol, together with the data from Hu et al. [35], which reported that 1 ng/mL recombinant leptin significantly stimulated estradiol release in cultured goose granulosa cells* in vitro*, may give an explanation, at least in part, for the early onset of puberty in girls with excess weight, which has been supported by Odongkara Mpora et al. [36], Castilho and Nucci [37], and our daily clinical work.

### 4.2. Associations of Kisspeptin with Pubertal Stages and Gender in Normal/Overweight/Obesity Status

In our present study, we firstly found that serum kisspeptin concentrations gradually increased from T2 to T5 stage and reached the highest levels in T5 stages in normal weight and obese/overweight boys. This change trend was similar to that of leptin in pubertal development girls. However, serum kisspeptin levels in prepubertal girls were lower and sharply increased to the highest level after the onset of puberty and then gradually decreased to the prepubertal levels in T5 stage in overall girls and obese/overweight girls. This change trend was also similar to that of leptin in pubertal development boys. This was in accordance with the study performed by Ma et al. [18], who found that serum kisspeptin concentrations of healthy southern Chinese adolescent girls were also the highest in pubertal stage 2. Serum kisspeptin levels were significantly higher in girls with central precocious puberty (CPP) from different countries than those in age-matched prepubertal controls [38–40]. Kisspeptin was positively related to peak LH and peak LH/FSH ratio during GnRH stimulation test, and there was a significant decline in the kisspeptin levels of girls with CPP after pubertal suppression [39]. In our present study, we also found that serum kisspeptin levels were positively correlated with serum LH and FSH levels in both boys and girls, and FSH was an independent contributor to circulating kisspeptin levels in girls. Young et al. [12] found that continuous kisspeptin infusion restored gonadotropin pulsatility in patients with loss-of-function mutations in NKB (TAC3) or its receptor (TAC3R). Jayasena et al. found that a single injection of kisspeptin-54 temporarily increased circulating LH levels and LH pulsatility in healthy women [41]. All these findings suggest that kisspeptin may play an important role in the regulation of puberty by the interaction with LH and FSH.

Pita et al. [42] found that obese prepubertal girls had higher circulating kisspeptin levels than normal weight prepubertal girls. Our study also showed that obese/overweight adolescent girls had notably higher serum kisspeptin concentrations than normal weight adolescent girls, and they were positively correlated with BMI, WC, and weight in girls. However, there were no significant differences in serum kisspeptin levels between normal weight boys and obese/overweight boys, and serum kisspeptin levels were positively correlated with leptin levels in girls, not in boys. Consistent with our observation of sexual dimorphism in kisspeptin levels of children and adolescents, studies performed by Young et al. [12] and Hrabovszky [13] found that the effects of kisspeptin-10 on gonadotropins secretion showed sexual dimorphism in humans, and preoptic kisspeptin neurons played a critical sex-specific role in positive estrogen feedback in female rodents. The similar phenomenon was observed in the literature, which showed that kisspeptin immunoreactivity in the arcuate nucleus was significantly higher in female mice than in male mice during puberty [43]. In addition, Tolson et al. [15] also found that Kiss1R knockout caused impaired glucose tolerance and obesity only in adult female mice, while Kiss1R knockout adult male mice showed no significant difference in body weight and glucose regulation when compared to the wild-type. These findings, together with our results, suggest that kisspeptin may exhibit the sexual dimorphism in regulating pubertal development.

### 4.3. Associations of Leptin and Kisspeptin with Lipid Profiles in Children and Adolescents

It is well established that leptin is the adiposity hormone and is closely linked with obesity and its related disorders [2]. Hlavaty et al. [44] found that leptin levels were associated with plasma palmitoleic acid contents in obese Czech adolescents. Yamborisut et al. [45] also showed that leptin concentrations were significantly associated with serum TG and waist circumference in obese Thai children. In parallel with these results, we also observed leptin levels were positively correlated with LDL-C and TG and negatively related to HDL-C in both boys and girls.

Lastly, we found that serum kisspeptin levels were negatively related to HDL-C, and HDL-C was an independent contributor to serum kisspeptin levels in boys. Wu et al. [46, 47] reported that the treatment with kisspeptin-10 significantly increased the levels of TG and LDL-C in primary cultured hepatocytes of chickens and kisspeptin-10 injected* in vivo* could markedly increase lipid anabolism in liver of birds, whereas Overgaard et al. [48] showed a strong negative correlation between the number of kisspeptin neurons in the arcuate nucleus of male rats and plasma triglyceride concentrations. Only a few of the literatures about the relationship between kisspeptin and serum lipids levels were reported up to now. Therefore, further researches need to be done to elucidate the issue.

In summary, in our present study, we firstly investigated the serum kisspeptin levels from the T1 to T5 stage in both adolescent boys and girls in such a larger sample. Our results demonstrated that serum kisspeptin levels were notably changed in different pubertal stages and nutritional states in Northern Chinese children and adolescents. The change characteristics seem to be different in boys and girls. Serum kisspeptin in girls firstly rose at pubertal stage 2 and then fell significantly to prepubertal levels at pubertal stage 5, while kisspeptin in boys gradually rose and reached the highest levels at pubertal stage 5. Kisspeptin levels in obese/overweight girls were significantly higher than those in normal weight girls, and they were positively correlated with weight, BMI, WC, FSH, and LH in all boys and girls. Our results lay the foundation for further exploration of kisspeptin and its role in pubertal development, especially for the sexual development disorders. However, this is a cross-sectional study. The conclusion of causal relationship between kisspeptin, pubertal stage, and nutrient status cannot be drawn in the present study. Many more detailed works need to be done in the future.

## Figures and Tables

**Table 1:** The general characteristics of overall boys and girls in different pubertal stages (mean ± SE).

	T1	T2	T3	T4	T5
Boys					
Number	102	44	63	73	75
Age (yr)	8.9 ± 0.1	12.0 ± 0.2^a^	13.6 ± 0.1^ab^	15.0 ± 0.2^abc^	16.2 ± 0.2^abcd^
Height (cm)	133.0 ± 0.9	148.0 ± 1.0^a^	161.0 ± 1.0^ab^	167.1 ± 0.7^abc^	170.6 ± 0.6^abcd^
Weight (kg)	34.3 ± 1.1	41.1 ± 1.2^a^	51.6 ± 1.4^ab^	60.1 ± 1.4^abc^	65.2 ± 1.5^abcd^
WC (cm)	64.8 ± 1.1	66.0 ± 1.3	66.8 ± 1.3	71.8 ± 1.1^abc^	74.6 ± 1.1^abc^
BMI (kg/m^2^)	19.0 ± 0.4	18.7 ± 0.5	19.8 ± 0.4	21.5 ± 0.4^abc^	22.4 ± 0.4^abc^
ALT (U/L)	16.62 ± 0.93	14.07 ± 0.77	13.37 ± 0.63	15.55 ± 0.98	20.92 ± 3.38
AST (U/L)	24.52 ± 0.53	22.89 ± 0.61	19.49 ± 0.51^ab^	19.90 ± 0.67^ab^	20.48 ± 1.35^a^
Urea (mmol/L)	4.16 ± 0.09	4.10 ± 0.12	4.50 ± 0.15	4.95 ± 0.15^ab^	4.83 ± 0.13^ab^
Cr (*μ*mol/L)	53.73 ± 0.69	59.98 ± 1.05^a^	67.87 ± 1.21^ab^	74.95 ± 1.02^abc^	79.99 ± 1.10^abcd^
TC (mmol/L)	3.82 ± 0.07	3.86 ± 0.10	3.45 ± 0.07^ab^	3.44 ± 0.05^ab^	3.41 ± 0.06^ab^
TG (mmol/L)	0.78 ± 0.04	0.74 ± 0.06	0.74 ± 0.05	0.76 ± 0.04	0.84 ± 0.05
HDL-C (mmol/L)	1.53 ± 0.03	1.59 ± 0.04	1.40 ± 0.04^ab^	1.34 ± 0.03^ab^	1.28 ± 0.03^abc^
LDL-C (mmol/L)	2.10 ± 0.05	2.05 ± 0.09	1.80 ± 0.06^ab^	1.83 ± 0.04^ab^	1.85 ± 0.05^ab^
LH (U/L)	0.14 ± 0.02	0.85 ± 0.08^a^	1.41 ± 0.07^ab^	1.78 ± 0.09^abc^	1.91 ± 0.10^abc^
FSH (U/L)	1.08 ± 0.01	2.39 ± 0.17^a^	3.51 ± 0.22^ab^	3.58 ± 0.19^ab^	2.75 ± 0.13^abcd^
T (ng/mL)	0.17 ± 0.07	0.77 ± 0.10^a^	2.02 ± 0.10^ab^	2.84 ± 0.11^abc^	3.04 ± 0.12^abc^
Leptin (ng/mL)	0.88 ± 0.09	0.65 ± 0.08	0.59 ± 0.07	0.66 ± 0.10	0.65 ± 0.08
Kisspeptin (pg/mL)	314.1 ± 14.0	309.3 ± 19.4	342.6 ± 17.5	396.7 ± 16.4^abc^	413.8 ± 14.5^abc^

Girls					
Number	78	24	41	47	100
Age (yr)	7.3 ± 0.1	8.9 ± 0.2^a^	10.7 ± 0.3^ab^	11.0 ± 0.2^abc^	14.9 ± 0.2^abcd^
Height (cm)	124.3 ± 0.8	130.8 ± 1.3^a^	143.3 ± 1.4^ab^	146.1 ± 1.1^abc^	157.0 ± 0.6^abcd^
Weight (kg)	25.7 ± 0.6	28.5 ± 1.2	35.6 ± 1.2^ab^	39.0 ± 0.9^ab^	56.8 ± 1.3^abcd^
WC (cm)	56.8 ± 0.8	58.7 ± 1.5	60.5 ± 1.0^a^	63.4 ± 0.9^a^	71.3 ± 1.0^abcd^
BMI (kg/m^2^)	16.5 ± 0.3	16.6 ± 0.5	17.2 ± 0.4	18.2 ± 0.4^a^	23.0 ± 0.5^abcd^
ALT (U/L)	13.91 ± 0.66	12.92 ± 0.61	13.19 ± 0.76	12.57 ± 0.74	13.43 ± 0.73
AST (U/L)	25.14 ± 0.56	23.38 ± 0.55	21.12 ± 0.78^a^	19.89 ± 0.61^ab^	17.75 ± 0.42^abcd^
Urea (mmol/L)	3.88 ± 0.11	3.82 ± 0.15	3.69 ± 0.13	3.64 ± 0.12	4.18 ± 0.11^acd^
Cr (*μ*mol /L)	47.91 ± 0.92	54.42 ± 1.09^a^	55.07 ± 1.19^a^	55.00 ± 0.96^a^	65.47 ± 0.76^abcd^
TC (mmol/L)	3.89 ± 0.06	3.82 ± 0.12	3.91 ± 0.10	3.67 ± 0.09	3.93 ± 0.07^d^
HDL-C (mmol/L)	1.58 ± 0.03	1.58 ± 0.07	1.54 ± 0.05	1.52 ± 0.04	1.45 ± 0.03^a^
LDL-C (mmol/L)	2.12 ± 0.05	2.05 ± 0.11	2.13 ± 0.08	1.94 ± 0.07	2.18 ± 0.06^d^
LH (U/L)	0.08 ± 0.01	0.14 ± 0.07	1.35 ± 0.22^ab^	1.49 ± 0.18^ab^	3.21 ± 0.20^abcd^
FSH (U/L)	1.54 ± 0.10	1.80 ± 0.19	3.72 ± 0.30^ab^	3.93 ± 0.25^ab^	4.00 ± 0.17^ab^
E2 (pg/mL)	7.78 ± 0.77	10.50 ± 1.50	30.19 ± 3.39^ab^	32.96 ± 2.82^ab^	59.13 ± 3.90^abcd^
Leptin (ng/mL)	0.79 ± 0.09	0.83 ± 0.11	0.93 ± 0.13	1.28 ± 0.13^a^	2.33 ± 0.11^abcd^
Kisspeptin (pg/mL)	299.2 ± 13.5	363.3 ± 26.6^a^	316.9 ± 21.3	299.9 ± 15.1^b^	327.9 ± 15.4

^a^Compared with pubertal stage 1, *P* < 0.05; ^b^compared with pubertal stage 2, *P* < 0.05; ^c^compared with pubertal stage 3, *P* < 0.05; ^d^compared with pubertal stage 4, *P* < 0.05.

WC, waist circumference; BMI, body mass index; ALT, alanine aminotransferase; AST, aspartate aminotransferase; Cr, creatinine; TC, total cholesterol; TG, triglycerides; HDL-C, high-density lipoprotein cholesterol; LDL-C, low-density lipoprotein cholesterol; LH, luteinizing hormone; FSH, follicle-stimulating hormone; T, testosterone; E2, estradiol.

**Table 2:** Correlation analysis of serum leptin and kisspeptin levels with other clinical items.

	Boys		Girls	
	Leptin	Kisspeptin	Leptin	Kisspeptin
BMI	0.659^a^	0.095^a^	0.780^a^	0.130^a^
WC	0.625^a^	0.106^a^	0.799^a^	0.162^a^
Weight	0.368^a^	0.205^a^	0.781^a^	0.111^a^
HDL-C	−0.151^a^	−0.221^a^	−0.243^a^	−0.094
LDL-C	0.267^a^	0.014	0.157^a^	0.036
TG	0.224^a^	0.081	0.252^a^	0.059
E2	—	—	0.442^a^	0.049
T	−0.193^a^	0.245^a^	—	—
FSH	−0.075	0.139^a^	0.200^a^	0.175^a^
LH	−0.101^a^	0.224^a^	0.368^a^	0.127^a^
Age	−0.055	0.277^a^	0.584^a^	0.064
Kisspeptin	0.008	—	0.140^a^	—

^a^ *P* < 0.05.

WC, waist circumference; BMI, body mass index; TG, triglycerides; HDL-C, high-density lipoprotein cholesterol; LDL-C, low-density lipoprotein cholesterol; LH, luteinizing hormone; FSH, follicle-stimulating hormone; T, testosterone; E2, estradiol.

**Table 3:** Regression analysis of serum leptin and kisspeptin levels with other clinical items.

	*R*	*R* ^2^	Factors	Standard partial regression coefficient	*P* value
Leptin					
Boys	0.750	0.562	BMI	0.491	<0.001
			T	−0.346	<0.001
			WC	0.236	0.005
Girls	0.834	0.696	WC	0.529	<0.001
			BMI	0.214	0.009
			E2	0.105	0.006

Kisspeptin					
Boys	0.329	0.108	HDL-C	−0.161	0.003
Girls	0.225	0.051	FSH	0.186	0.002

WC, waist circumference; BMI, body mass index; FSH, follicle-stimulating hormone; T, testosterone; E2, estradiol.
